# Phyllodulcin, a Natural Sweetener, Regulates Obesity-Related Metabolic Changes and Fat Browning-Related Genes of Subcutaneous White Adipose Tissue in High-Fat Diet-Induced Obese Mice

**DOI:** 10.3390/nu9101049

**Published:** 2017-09-21

**Authors:** Eunju Kim, Soo-Min Lim, Min-Soo Kim, Sang-Ho Yoo, Yuri Kim

**Affiliations:** 1 Department of Nutritional Science and Food Management, Ewha Womans University, Seoul 03760, Korea; eunju831@naver.com (E.K.); arlenalim@naver.com (S.-M.L.); 2 Department of Food Science and Biotechnology, and Carbohydrate Bioproduct Research Center, Sejong University, 209 Neungdong-ro, Gwangjin-gu, Seoul 05006, Korea; main0789@naver.com (M.-S.K.); shyoo@sejong.ac.kr (S.-H.Y.)

**Keywords:** phyllodulcin, obesity, high fat diet, fat browning, hypothalamus, adipogenesis, subcutaneous fat, BDNF-TrkB

## Abstract

Phyllodulcin is a natural sweetener found in *Hydrangea macrophylla* var. *thunbergii*. This study investigated whether phyllodulcin could improve metabolic abnormalities in high-fat diet (HFD)-induced obese mice. Animals were fed a 60% HFD for 6 weeks to induce obesity, followed by 7 weeks of supplementation with phyllodulcin (20 or 40 mg/kg body weight (b.w.)/day). Stevioside (40 mg/kg b.w./day) was used as a positive control. Phyllodulcin supplementation reduced subcutaneous fat mass, levels of plasma lipids, triglycerides, total cholesterol, and low-density lipoprotein cholesterol and improved the levels of leptin, adiponectin, and fasting blood glucose. In subcutaneous fat tissues, supplementation with stevioside or phyllodulcin significantly decreased mRNA expression of lipogenesis-related genes, including CCAAT/enhancer-binding protein α (*C/EBPα*), peroxisome proliferator activated receptor γ (*PPARγ*), and sterol regulatory element-binding protein-1C (*SREBP-1c*) compared to the high-fat group. Phyllodulcin supplementation significantly increased the expression of fat browning-related genes, including PR domain containing 16 (*Prdm16*), uncoupling protein 1 (*UCP1*), and peroxisome proliferator-activated receptor γ coactivator 1-α (*PGC-1α*), compared to the high-fat group. Hypothalamic brain-derived neurotrophic factor-tropomyosin receptor kinase B (BDNF-TrkB) signaling was upregulated by phyllodulcin supplementation. In conclusion, phyllodulcin is a potential sweetener that could be used to combat obesity by regulating levels of leptin, fat browning-related genes, and hypothalamic BDNF-TrkB signaling.

## 1. Introduction

The worldwide prevalence of obesity has more than doubled since 1980. According to the World Health Organization (WHO) in 2014, more than 1.9 billion adults were overweight and over 600 million were obese [1]. Obesity involves alterations in hormone production and metabolism, and it is a condition that results from an imbalance between energy intake and energy output. Genetic, environmental, and behavior risk factors can also contribute to obesity [2,3,4]. Intake of high-energy foods that contain large amounts of fats and sugars has dramatically increased over the past several decades [3,5]. This change in dietary habits is thought to be a key driver of the increasing prevalence of obesity. 

The main feature of obesity is fat accumulation. Adipose tissues exist in two types: white adipose tissue (WAT) stores energy as triglycerides (TGs), whereas brown adipose tissue (BAT) is the main site of energy consumption by activating thermogenesis [6]. The amount of BAT and the browning of subcutaneous fat have been associated with protection against metabolic diseases, such as obesity, diabetes, and dyslipidemia by suppressing excessive metabolic fat accumulation [7]. On the other hand, a subset of WAT can be changed into brown-like adipocytes, called “brite adipocytes” or “beige adipocytes” [8]. Changes in subcutaneous fat browning can strongly affect thermogenesis and glucose homeostasis [9,10]. The browning process includes the induction of uncoupling protein 1 (*UCP1*) and the expression of genes related to uncoupled respiration and heat production [8].

Brain-derived neurotrophic factor (BDNF) is an influential modulator of neuronal development and synaptic function. Recently, BDNF was reported to play a role in energy regulation [11,12]. BDNF, which is translated from the *Bdnf* mRNA of a long 3′ untranslated region (UTR) in the hypothalamus, is essential for leptin mediated regulation of body weight (b.w.) and energy balance [13]. Browning of subcutaneous fat was previously shown to be markedly linked to BDNF expression and leptin sensitivity in the hypothalamus [14]. Leptin sensitivity was shown to be decreased in high-fat diet compared to low-fat diet induced mice, due to increases in fat storage and plasma leptin levels [15]. Low leptin sensitivity caused impairment of energy metabolism, such as hyperglycemia and dyslipidemia [16]. Therefore, enhancing both BDNF expression and leptin sensitivity by promoting browning of subcutaneous fat may be a good strategy to improve energy metabolism. In addition, BDNF upregulates peroxisome proliferator-activated receptor γ coactivator 1-α (*PGC-1α*), a major regulator of mitochondrial biogenesis [11], and activates tropomyosin receptor kinase B (TrkB), which can lead to phosphorylation of phosphatidylinositol-3 kinase (PI3K) and extracellular signal-regulated kinase (ERK) [11]. Therefore, enhancing BDNF expression in the hypothalamus by promoting fat browning-related gene expression in subcutaneous fat may represent a potential mechanism by which obesity-related metabolic disorders could be improved.

Increased sugar consumption is one of the main risk factors for obesity and metabolic diseases. High intake of refined grains or added sugars influences total energy intake and blood glucose levels, leading to metabolic changes and increased body fat accumulation [17,18]. Although low-calorie artificial sweeteners can help to decrease blood glucose levels and inhibit metabolic disorders [18], the effects of artificial sweeteners on obesity and related metabolic disorders remain controversial. For example, one study showed that stevia preload reduced blood glucose and insulin levels compared to sucrose preload, suggesting that stevia may help to regulate blood glucose compared to sucrose consumption [19]. In contrast, the non-calorie artificial saccharine induced glucose intolerance by causing gut microbiota dysbiosis and abnormalities of host glucose metabolism [20]. Therefore, the development of the new effective natural sugars with few side-effects is a good strategy to improve the treatment of metabolic disorders.

Hydrangea (*Hydrangea macrophylla* var. *thunbergii*) has been utilized as a dried leaf tea in Asian countries [21]. Its extracts possess anti-diabetic, anti-ulcer, and anti-fungal effects [22,23]. Phyllodulcin is one of the isocumarin derivatives in Hydrangea and is a well-known natural sweetener that is 600 to 800 times sweeter than sucrose [24,25]. Phyllodulcin acts as a non-selective phosphodiesterase inhibitor and exerts anti-allergic effects by suppressing lymphocyte activation [26,27]. However, there is lack of evidence about the anti-obesity effects of phyllodulcin. Therefore, the aim of the present study was to determine whether phyllodulcin could be beneficial for hyperglycemia and dyslipidemia by activating fat browning-related genes of subcutaneous fat and regulating BDNF signaling in the hypothalamus in a mouse model of high-fat diet (HFD)-induced obesity.

## 2. Materials and Methods 

### 2.1. Materials and Sample Preparation

Phyllodulcin was extracted from hydrangea leaves by following the method reported previously [28]. Briefly, leaves were harvested in October 2013 (Sugukmiso Co., Daegu, Korea) and freeze-dried until phyllodulcin extraction. Dried hydrangea leaves were drenched in distilled water for 12 h. Phyllodulcin was extracted by using 75% (*v*/*v*) ethanol. The extract of hydrangea leaves was passed through a mixed-bed ion exchanger column. Phyllodulcin was isolated by using a preparative high-performance liquid chromatography system that was equipped with an autosampler and photodiode array (PDA)-UV detector (Thermo-Finnigan Surveyor, Thermo Scientific, Sunnyvale, CA, USA) for purifying phyllodulcin. Final purity and yield of phyllodulcin were 97% and 2.12% (dry basis), respectively [28]. Purified phyllodulcin was kept in an auto-desiccator (Sanpla Dry Keeper, Sanplatec Corp, Osaka, Japan) until it was used for experiments. 

### 2.2. Animals and Diet

Animals used in this study were 5-week-old male C57BL/6 mice (Central Lab Animal Inc., Seoul, Korea) with similar b.w. (18–20 g) at the beginning of the study. Mice were housed in polycarbonate cages under a 12 h/12 h light/dark cycle with controlled temperature (22 ± 2 °C) and humidity (50% ± 5%). Mice were provided ad libitum access to sterile water. 

Control (Ctrl) group mice received the American Institute of Nutrition 93G (AIN93G) diet for the duration of the study. All other mice received a 60% HFD (Unifaith Inc., Seoul, Korea) for 6 weeks and were randomly assigned to one of four groups (*n* = 12 mice per group). Mice in these four groups received the 60% HFD for 7 additional weeks, which was supplemented with stevioside at 40 mg/kg b.w./day (SVS), phyllodulcin at 20 mg/kg b.w./day (P 20), or phyllodulcin at 40 mg/kg b.w./day (P 40). Among the 58 total animals used in this study (Ctrl, *n* = 10; HF, SVS, P 20, and P 40, *n* = 12 each), one mouse in the HF group died during vehicle gavage treatment. Therefore, a total of 57 animals were analyzed. Phyllodulcin and stevioside supplementation were given by gavage. Stevioside, a commercially available sweetener, was used as a positive control. 

Food intake and b.w. of mice were monitored twice a week throughout the experimental period. At the end of feeding for 13 weeks, all mice were sacrificed. Blood was immediately collected from the abdominal vein and stored in ehylenediaminetetraacetic acid (EDTA) tubes for determining plasma parameters after centrifugation at 1300× *g* for 10 min. Hypothalamus and subcutaneous fat were dissected and stored at −80 °C for further analyses. All experimental protocols were approved by the Institutional Animal Care and Use Committee of Ewha Womans University (No. 16-035).

### 2.3. Biochemical Analysis of Blood Samples

Plasma levels of TGs (#AM157S-K) and total cholesterol (TC, #AM203-K) were determined by relevant commercial kits (Asan Pharmaceutical Co., Seoul, Korea). Low-density lipoprotein (LDL) cholesterol levels were estimated by the formula: LDL cholesterol = TC − (TG/5 + high-density lipoprotein cholesterol (HDL-C)). Plasma levels of leptin (#90030) and adiponectin (#80569) were analyzed by using commercially available kits (Crystal Chem, Downers Grove, IL, USA). On the final day of the experiment, fasting blood glucose (FBG) concentrations were measured from the tail vein with a glucometer (ACCU-CHEK Active, Roche, Mannheim, Germany). All profiles were analyzed according to the manufacturers’ instructions.

### 2.4. Western Blotting Analysis

Immunoblotting analysis was performed as previously described with minor modifications [29]. In brief, total protein was extracted from the hypothalamus by using protein extract solution (PRO-PREP, Intron Biotechnology, Seoul, Korea). Protein levels of samples were measured by colorimetric protein assay kits (BIO-RAD, Hercules, CA, USA). Denatured proteins were separated with 6–15% sodium dodecyl sulfate-polyacrylamide gel electrophoresis, and transferred to polyvinylidene difluoride membranes, which were blocked with 5% bovine serum albumin or 5% low-fat milk in Tris-buffered saline with Tween 20 for 1 h. Membranes were incubated overnight at 4 °C with antibodies against BDNF (Abcam, Cambridge, UK), TrkB, PI3K, phospho-PI3K, ERK (1/2), and phospho-ERK (1/2) (Cell Signaling, Danvers, MA, USA) followed by incubation for 1 h at room temperature with suitable horseradish peroxidase conjugated secondary antibody (Santa Cruz Biotechnology, Dallas, TX, USA). Immunoblotting signals were visualized with an enhanced chemiluminescence reagent (Animal Genetics Inc., Suwon, Gyeonggi-do, Korea). α-tubulin (Sigma Aldrich, St. Louis, MO, USA) was used as the loading control.

### 2.5. RNA Isolation and Real-Time Polymerase Chain Reaction (PCR) Analysis

Total RNA was isolated from subcutaneous fat by using TRIzol reagent (Invitrogen, Carlsbad, CA, USA), cDNA was synthesized by using the RevertAid First Strand cDNA Synthesis Kit (Thermo Fisher Scientific, Waltham, MA, USA). Quantitative real-time PCR was conducted on a Rotor-Gene Q instrument system (Qiagen, Frederick, MD, USA) by using SYBR Green PCR kits (Qiagen). The amplification step was performed with the following conditions: initiation step at 95 °C for 5 min and 40 cycles of denaturation at 94 °C for 15 s, followed by annealing at 55 °C for 30 s, and extension at 70 °C for 30 s. Relative mRNA level was quantified by the 2^−ΔΔCT^ method. PCR sequences for various genes are presented in Table 1. Glyceraldehyde-e-phosphate dehydrogenase (GAPDH) was used as an internal control.

### 2.6. Statistical Analyses

Statistical analyses were performed with GraphPad Prism (GraphPad Software, Inc., La Jolla, CA, USA). Data were presented as the mean ± standard error of the mean (SEM). Significant differences among groups were determined by using one-way analysis of variance (ANOVA) with Tukey’s post hoc tests. Pearson’s correlation was used to analyze correlations between the weight of subcutaneous fat and the levels of leptin or adiponectin. A *p*-value of less than 0.05 was considered to indicate statistical significance.

## 3. Results

### 3.1. B.W., Food Intake, and Fat and Liver Weight

During the 13-week experimental period, b.w., food intake, and organ weight were measured for all mice (Table 2). After 7 weeks of supplementation, final b.w. was higher in the HF group than in the Ctrl group (*p* < 0.001). Supplementation with stevioside or phyllodulcin did not affect b.w. changes compared to the HF group. Body mass index (BMI) tended to be lower in the phyllodulcin supplementation groups than the BMI of the HF group, but this difference was not significant. Food intake was lower in the HF group than in the Ctrl group (*p* < 0.001). Stevioside supplementation decreased food intake compared to the HF group (*p* < 0.001), but phyllodulcin supplementation did not have the same effect. 

The total fat (subcutaneous, mesenteric, epidydimal, and perirenal fat) weight was higher in the HF group than in the Ctrl group (*p* < 0.01). Phyllodulcin supplementation groups tended to have lower total fat mass values than the HF group, but these differences were not significant. Subcutaneous and mesenteric fat weights were higher in the HF group than in the Ctrl group (all *p* < 0.001). Whereas subcutaneous fat weight was decreased in the P 40 group (*p* < 0.05), mesenteric fat weight was not affected by phyllodulcin supplementation. 

### 3.2. Plasma Biochemical Profiles

Concentrations of plasma lipids (TGs, TC, and LDL), FBG, leptin, and adiponectin were presented in Table 3. The levels of plasma TG, TC, and LDL were significantly increased in the HF group compared to the Ctrl group (*p* < 0.05 for TGs and LDL, *p* < 0.001 for TC). These levels were lower in both phyllodulcin supplementation groups than those in the HF group, with a decrease of 14.7% for TGs (*p* < 0.01), 14.5% for TC (*p* < 0.05), and 42.3% for LDL (*p* < 0.01) in the P 40 group. 

The FBG level was significantly higher in the HF group than in the Ctrl group (*p* < 0.001). The FBG level was 34.0% lower in the SVS group, 33.4% lower in the P 20 group, and 37.0% lower in the P 40 group than in the HF group (all *p* < 0.001). The leptin level was significantly higher in the HF group than in the Ctrl group (*p* < 0.001). Compared to the HFD-fed group without supplementation, 40 mg/kg b.w./day phyllodulcin supplementation inhibited the leptin level by 17.6% (*p* < 0.05). The adiponectin level was lower in the HF group than in the Ctrl group (*p* < 0.001), while both dosages of phyllodulcin supplementation increased the adiponectin level compared to the HF group (*p* < 0.05 for P 20, *p* < 0.01 for P 40). Stevioside did not affect the level of adiponectin.

### 3.3. Correlation between Weight of Subcutaneous Fat and Levels of Leptin and Adiponectin

**Figure 1:**
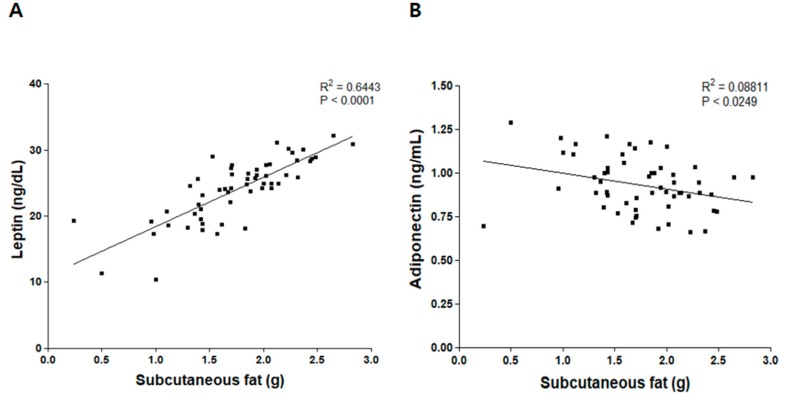
Association between subcutaneous fat and levels of leptin and adiponectin. Pairwise scatter plots for correlations between subcutaneous fat and (**A**) plasma leptin level or (**B**) plasma adiponectin level.

Subcutaneous fat mass has been positively associated with leptin concentration [30,31]. We confirmed this association in our HFD-induced obesity model (Figure 1). Scatter plots were used to view correlations between subcutaneous fat weight and levels of plasma leptin and adiponectin. Subcutaneous fat weight was positively correlated with leptin level (*p* < 0.0001, *R*^2^ = 0.6443), but negatively correlated with adiponectin level (*p* < 0.0249, *R*^2^ = 0.08811).

### 3.4. Expression of Genes Related to Adipogenesis, Lipogenesis, and Browning in Subcutaneous Fat

**Figure 2:**
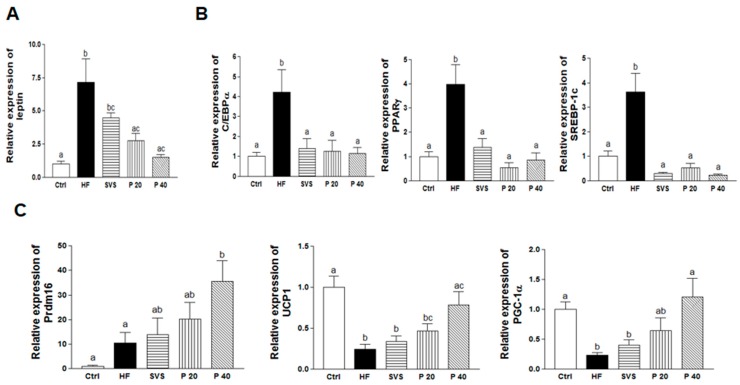
Expressions of *leptin*, adipogenesis, lipogenesis, and browning-related genes in subcutaneous fat. Real-time PCR was performed to analyze adipogenesis, lipogenesis, and browning-related genes in subcutaneous fat. (**A**) *leptin*, (**B**) (left) *C/EBPα*, (middle) *PPARγ*, and (right) *SREBP-1C* mRNA expressions; (**C**) (left) *Prdm16*, (middle) *UCP1*, and (right) *PGC-1α* mRNA expressions. Values are shown as the means ± SEM. All data were analyzed with one-way ANOVA and Tukey’s post hoc tests. GADPH was used as the loading control. Ctrl, non-obese control; HF, high-fat diet-induced obese mice; SVS, high-fat diet-induced obese mice that received 40 mg/kg b.w. stevioside/day; P 20, high-fat diet-induced obese mice that received 20 mg/kg b.w./day phyllodulcin; P 40, high-fat diet-induced obese mice that received 40 mg/kg b.w./day phyllodulcin. *C/EBPα*, CCAAT/enhancer-binding protein alpha; *PPARγ*, peroxisome proliferator activated receptor γ; *SREBP-1c*, sterol regulatory element-binding protein-1C; Prdm16, PR domain containing 16; *UCP1*, uncoupling protein 1; *PGC-1α*, peroxisome proliferator-activated receptor γ coactivator 1-α; GAPDH, glyceraldehyde-3-phosphate dehydrogenase.

Expressions of *leptin* mRNA were confirmed to investigate leptin synthesis in subcutaneous fat tissues (Figure 2A). Levels of *leptin* mRNA were upregulated in the HF group compared to the Ctrl group (*p* < 0.001). However, phyllodulcin supplementation significantly downregulated the levels of *leptin* mRNA compared to the HF group by 61.3% and 79.2%, respectively (*p* < 0.01 for P 20, *p* < 0.001 for P 40), whereas stevioside supplementation did not affect levels of *leptin* mRNA in subcutaneous fat tissues.

Expressions of genes related to adipogenesis and lipogenesis, such as CCAAT/enhancer-binding protein α (*C/EBPα*), peroxisome proliferator activated receptor γ (*PPARγ*), and sterol regulatory element-binding protein-1C (*SREBP-1c*) were analyzed to investigate the effect of phyllodulcin on adipogenesis and lipogenesis in subcutaneous fat (Figure 2B). Expression levels of all analyzed adipogenic and lipogenic genes were upregulated in the HF group compared to the Ctrl group (*p* < 0.01 for *C/EBPα*, *p* < 0.001 for *PPARγ* and *SREBP-1c*). Stevioside and phyllodulcin supplementation significantly lowered the HFD-induced upregulation of these genes. In particular, 40 mg/kg b.w./day phyllodulcin supplementation markedly suppressed expression of *C/EBPα* by 72.7% (*p* < 0.01), *PPARγ* by 78.7% (*p* < 0.001), and *SREBP-1c* by 93.6% (*p* < 0.001) compared to the HF group. 

In addition, the expression levels of genes related to fat browning, including PR domain containing 16 (*Prdm16*), uncoupling protein 1 (*UCP1*), and peroxisome proliferative activated receptor γ coactivator 1 α (*PGC-1α*), were analyzed to investigate the effect of phyllodulcin in subcutaneous fat browning (Figure 2C). The mRNA expression of *Prdm16*, which mediates conversion of white to beige fat, was increased 3.4-fold in the P 40 group compared to the HF group (*p* < 0.05). The mRNA expression levels of two other fat conversion mediators, *UCP1* and *PGC-1α*, were significantly increased by 3.24- and 5.23-fold, respectively, in the P 40 group compared to the HF group (all *p* < 0.01). These results indicate that phyllodulcin suppressed adipogenesis and increased browning of white to beige fat. 

### 3.5. Brain Weight and BDNF Signaling in Hypothalamus

**Figure 3:**
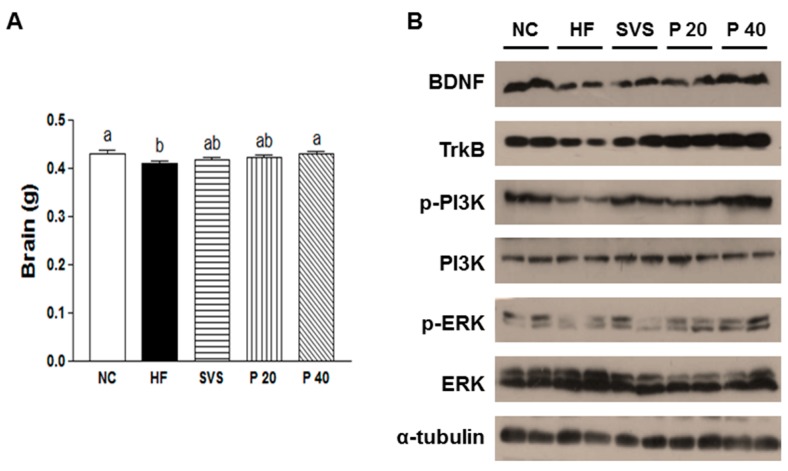
Brain weight (b.w.) and BDNF signaling in hypothalamus. (**A**) b.w.; (**B**) Expression of BDNF signaling, including BDNF, TrkB, PI3K, and ERK in hypothalamus. Hypothalamus organs from five to six mice were pooled, and individual pooled samples are shown. Values are shown as the means ± standard error or the mean (SEM). All data were analyzed with one-way ANOVA and Tukey’s post hoc tests. α-tubulin was used as the loading control. Ctrl, non-obese control; HF, high-fat diet-induced obese mice; SVS, high-fat diet-induced obese mice that received 40 mg/kg b.w. stevioside/day; P 20, high-fat diet-induced obese mice that received 20 mg/kg b.w./day phyllodulcin; P 40, high-fat diet-induced obese mice that received 40 mg/kg b.w./day phyllodulcin. BDNF, brain-derived neurotrophic factor, TrkB, tropomyosin receptor kinase B; PI3K, phosphatidylinositol-e kinase; ERK; extracellular signal-regulated kinases.

To understand hypothalamic regulation by phyllodulcin supplementation, brain weight and BDNF signaling in the hypothalamus tissue were analyzed (Figure 3). Brain weight was decreased in the HF group (*p* < 0.05), which confirmed the previously described slight brain atrophy of obese mice [32]. This reduction of brain weight was recovered in the P 40 group to the level of the Ctrl group (Figure 3A). Phyllodulcin supplementation upregulated protein expression levels of BDNF and TrkB and increased phosphorylation of PI3K and ERK (Figure 3B).

## 4. Discussion

In the present study, phyllodulcin supplementation improved the level of FBG and plasma lipid profiles, while reducing subcutaneous fat weight. In the mice supplemented with phyllodulcin, genes related to adipogenesis and lipogenesis were downregulated, while genes related to fat browning in subcutaneous fat were upregulated. Activation of the BDNF signaling pathway in the hypothalamus was also observed in the phyllodulcin-treated mice. These results suggest that phyllodulcin supplementation has the potential to be an effective alternative natural sweetener in obesity-related metabolic diseases.

In 2015, the WHO recommended that free and added sugars be reduced to less than 10% of the total energy intake for both adults and children, with further reduction of the intake of free sugars to less than 5% of the total energy intake [33]. The development of high-intensity sugar alternatives would be beneficial for individuals seeking to reduce their sugar intake and risk of obesity-related metabolic diseases. 

A previous study showed that obese women who used aspartame-sweetened foods and beverages for 16 weeks showed reduced b.w. [34]. Treatment with 10–20 mg/kg b.w. stevioside enhanced insulin sensitivity and antioxidant defense and improved blood glucose metabolism and adipose inflammation in diabetic and obese mice [35,36,37]. In the present study, phyllodulcin supplementation affected most of the indicators examined, and the results were similar to those observed with stevioside. However, phyllodulcin treatment induced a greater effect on the levels of leptin, adiponectin, and fat browning-related genes compared to stevioside. Thus, phyllodulcin appears to be a natural sweetener that is comparable to stevioside for obesity-related metabolic disorders, and further comparisons between the two sweeteners regarding other health benefits need to be investigated. 

Leptin is a 16 kDa protein that is primarily produced by adipocytes in proportion to the size of fat stores [38,39]. Plasma leptin levels increase in proportion to body fat mass and energy expenditure to sustain body fat stores by regulating food intake [38,40]. Plasma leptin levels were lower in the phyllodulcin treatment groups than in the HF group. BMI tended to be decreased by phyllodulcin supplementation, but this result was not significant. Plasma leptin levels are strongly associated with fat, especially subcutaneous fat, as it produces a greater amount of leptin than other types of fat, such as omental fat [30,31]. Here, supplementation of phyllodulcin decreased the weight of subcutaneous fat more than the weight of mesenteric fat. Subcutaneous fat weight was correlated positively with plasma concentrations of leptin, but negatively associated with plasma levels of adiponectin. Plasma leptin and adiponectin levels were previously found to be significantly increased and decreased, respectively, in obese subjects in proportion to the percent of body fat [41,42]. In addition, levels of *leptin* mRNA were highly correlated with adipocyte volume and weight [43,44]. In obese subjects, expressions of *leptin* mRNA were correlated with mRNA levels of adipogenesis genes, including *PPARγ* and *C/EBPα*, in intraperitoneal and extraperitoneal fat [45]. In particular, levels of *leptin* mRNA in subcutaneous fat were highly related with serum leptin level in high-fat diet induced obesity rats [46], and leptin secretion rates or mRNA levels of *leptin* in subcutaneous fats were highly correlated with the levels of serum leptin in women [47,48]. These indicate that leptin plays a role in regulating adipose tissue distribution [48]. In the present study, phyllodulcin supplementation downregulated the expression of *leptin* mRNA in subcutaneous fat compared to the HF group, which might be related with plasma leptin levels. 

Subcutaneous fat is more related to fat browning than other fats because beige adipocytes are especially abundant in the subcutaneous fat, especially the inguinal WAT. These adipocytes have clusters of UCP-1 expression with thermogenic capacity [49]. Similar to BAT, beige cells in WAT have high levels of mitochondria and express BAT specific genes, such as *UCP1* and *PGC-1α* [50]. Development of these thermogenic-capable cells in WAT improved resistance against metabolic diseases such as obesity [51]. In previous reports, leptin induced WAT browning and decreased adiposity in hypothalamic neurons [52]. Leptin increased adiponectin levels by chronic cold exposure-induced browning of subcutaneous fat through binding with accumulated M2 macrophages [53]. Taken together, these findings suggest that both leptin and adiponectin levels can affect subcutaneous fat and increase the amount of beige fat. 

*C/EBPα*, *PPARγ*, and *SREBP-1c* are major players in adipogenesis and lipogenesis. *C/EBPα* and *PPARγ* activate the early phase of adipogenesis. *PPARγ* is a first inducer of fat cell development and promotes adipogenesis by co-expression with C/EBPα [54,55,56]. *SREBP1c* is a major regulator of lipogenesis and lipid homeostasis by inducing *PPARγ* gene expression [54,57]. In the present study, phyllodulcin supplementation suppressed adipogenesis and lipogenesis in subcutaneous fat by downregulating *C/EBPα*, *PPARγ*, and *SREBP1c*.

*Prdm16*, a large zinc finger transcription factor, plays a crucial role in the development of beige adipocytes [58,59]. Prdm16 is abundant in subcutaneous fat and is involved in the “browning” of WAT. Mice with knock-down of Prdm16 develop obesity, insulin resistance, and increased levels of subcutaneous adipose tissues [59]. After Prdm16-mediated browning of WAT, beige adipocytes have the ability to change from a WAT to a BAT phenotype with UCP1-containing adipocytes [60]. In addition, Prdm16 activates brown fat differentiation in WAT preadipocytes and regulates other transcriptional factors, including *UCP1* and *PGC-1α* by direct binding [61]. 

Mitochondrial uncoupling proteins (UCPs) are key molecules in thermogenesis. Among the three known UCPs, UCP-1 is abundant and specific for BAT [62]. Inner mitochondrial membranes express high levels of UCP1, which mediates the release of electrons, resulting in thermogenesis [63]. *PGC-1α*, induced by cold exposure, turns on key components for thermogenesis in brown fat by activating the expression of *UCP1* [64]. *PGC-1α* increases the transcriptional activity of *UCP-1* in WAT and promotes differentiation towards brown fat [63]. 

Phyllodulcin supplementation upregulated the expression of fat browning and thermogenesis-related genes, such as *Prdm16*, *UCP1*, and *PGC-1α*. Previously, *Prdm16* was demonstrated to regulate other transcriptional factors, including *C/EBPs*, *PPARα*, *PPARγ, UCP1*, and *PGC-1α* [61,65,66]. In the present study, *Prdm16* was upregulated, whereas *C/EBPα* and *PPARγ* were downregulated, by phyllodulcin supplementation. These results may be due to the fact that C/EBPs and PPARγ are involved in the differentiation of both white and brown fat [67]; this possibility should be determined in a future study. In addition, it is important to mention that fat browning increases energy expenditure and leads to favorable effects on metabolism, including improvement of glucose homeostasis and dyslipidemia by utilizing blood glucose and lipids [51,68,69]. In the present study, phyllodulcin improved the levels of FBG and blood lipids, including TG, TC, and LDL. Taken together, these results suggest that phyllodulcin supplementation may reduce subcutaneous fat, leptin levels, and metabolic abnormalities, including those related to blood glucose and lipids, by regulating the expression of both lipogenesis-related genes and browning-related genes. After binding to its receptor in the hypothalamus, leptin initiates central regulation of energy homeostasis [70], playing critical roles in thermogenesis and b.w. control. Together, leptin and insulin promote the browning of WAT [52]. Leptin is linked to BDNF in the energy balance and activates long 3′UTR *Bdnf* mRNA in hypothalamic dendrites [13]. BDNF is a neuronal growth indicator that recently was implicated in the transformation from WAT to BAT [71]. BDNF promotes thermogenesis in the hypothalamus and regulates energy expenditure. Hypothalamic overexpression of BDNF activated genes related to brown fat, including *Prdm16* and *UCP1* [14,71]. Administration of BDNF increased thermogenesis, norepinephrine turnover, and *UCP1* gene expression in BAT and improved glucose metabolism in db/db mice [72]. 

The production and release of BDNF promote its binding to receptor TrkB and activate signaling in the hypothalamus, increasing energy expenditure by modulating hypothalamic neurons [11,14]. Activation of TrkB by BDNF promotes synaptic plasticity and cAMP response element-binding protein (CREB) phosphorylation by inducing the expression of *PGC-1α* [11,73]. Multiple post-translational modification signals are involved in the activation of TrkB by BDNF. PI3K and MAPK are the major signaling pathways to regulate cellular energy balance and neuronal survival by BDNF [73,74]. In particular, PI3K and ERK pathways are downstream of leptin in thermogenesis [75,76]. In the present study, expression levels of BDNF and TrkB and phosphorylation of PI3K and ERK were increased by phyllodulcin supplementation. These results indicate that phyllodulcin regulated the fat browning-related genes of subcutaneous fat through BDNF-TrkB signaling in mice with HFD-induced obesity. 

Several studies have shown that dietary factors can affect fat browning. For example, capsaicin, a major spicy ingredient in hot pepper, induced browning of WAT in HFD-fed rats [77]. Resveratrol, a natural polyphenol in grape skin, increased mitochondrial DNA content and upregulated mRNA expression of *UCP1* in primary mouse embryonic fibroblasts-derived WAT and 3T3-L1 preadipocytes [78,79]. Conjugated linoleic acid reduced fat levels by inducing fat browning in gonadal adipose tissue [80]. 

In the present study, the focus of the present study was to investigate the anti-obesity effect of phyllodulcin by analyzing obesity-related metabolic changes as well as other changes. Fat browning-related genes and BDNF signaling have been implicated in mediating one of the effects of phyllodulcin. One limitation of the study is that physiological measurements were not analyzed, although they would improve evaluation of the role of phyllodulcin and could confirm the beneficial effects of phyllodulcin. Thus, future studies that include physiological measurements are warranted. However, the present study provides the first evidence for an anti-obesity effect of a low-calorie sweetener on obesity-related metabolic changes by regulating WAT browning-related genes in an animal model.

## 5. Conclusions

**Figure 4:**
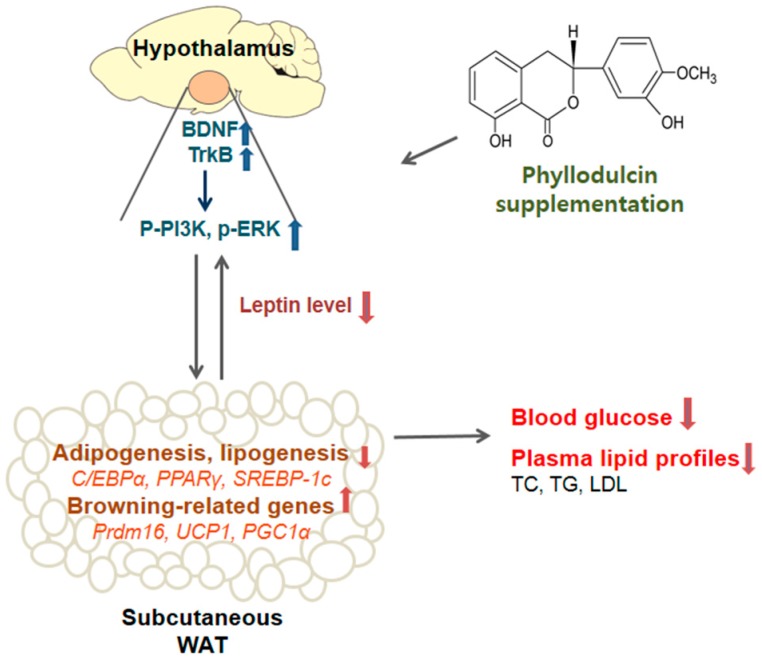
Potential mechanism of phyllodulcin. Supplementation with phyllodulcin decreased the amount of subcutaneous fat and the expression of adipogenesis and lipogenesis-related genes in mice with high-fat diet induced-obesity. This suppression may be due to regulation of plasma leptin level, fat browning-related genes of WAT, and BDNF signaling in the hypothalamus. Ultimately, supplementation of phyllodulcin improved blood lipid profiles and blood glucose levels. BDNF, brain-derived neurotrophic factor; *C/EBPα*, CCAAT/enhancer-binding protein alpha; ERK, extracellular signal-regulated kinases; LDL, low-density lipoprotein cholesterol; *PGC-1α*, peroxisome proliferator-activated receptor γ coactivator 1-α; PI3K, phosphatidylinositol-3 kinase; PPARγ, peroxisome proliferator activated receptor γ; *Prdm16*, PR domain containing 16; *SREBP-1c*, sterol regulatory element-binding protein-1C; TC, total cholesterol; TG, triglyceride; TrkB, tropomyosin receptor kinase B; *UCP1*, uncoupling protein 1; WAT, white adipose tissues.

Supplementation of HFD-induced obese mice with phyllodulcin decreased the weight of subcutaneous fat and the expression of adipogenesis and lipogenesis-related genes. Phyllodulcin improved fat browning-related genes of subcutaneous fat and regulated plasma leptin levels and related BDNF signaling in the hypothalamus. Ultimately, phyllodulcin supplementation improved the blood lipid profiles and glucose levels of mice (Figure 4). These results show the potential health benefits of using phyllodulcin as a therapeutic alternative sweetener in obesity-related metabolic diseases. Future studies investigating physiological changes of fat, thermogenesis activity-related mechanisms, and the energy metabolism of this process are warranted. Furthermore, long-term clinical trials are needed to confirm these results. 

## Figures and Tables

**Table 1:** The sequences of experimental primers used for q-PCR.

	Gene Symbol	Genbank ID	Forward Primer (5′ to 3′)	Reverse Primer (5′ to 3′)
Leptin	Lep	16486	TGACACCAAAACCCTCATCA	CTCAAAGCCACCACCTCTGT
C/EBPα	Cebpa	12606	CCAAGAAGTCGGTGGACAAGA	CGGTCATTGTCACTGGTCAACT
PPARγ	Pparg	19016	AAGAGCTGACCCAATGGTTG	TGAGGCCTGTTGTAGAGCTG
SREBP-1c	Srebf1	20787	TAGAGCATATCCCCCAGGTG	GGTACGGGCCACAAGAAGTA
Prdm16	Prdm16	70673	AGATGAACCAGGCATCCACT	TCTACGTCCTCTGGCTTTGC
UCP1	UCP1	22227	CCAAGCCAGGATGGTGAAC	CCAGCGGGAAGGTGATGATA
PGC-1α	Ppargc1a	19017	TCGAGCTGTACTTTTGTGGA	TCATACTTGCTCTTGGTGGA
GAPDH	Gapdh	14433	GCCTTCCGTGTTCCTACCC	TGCCTGCTTCACCACCTT

C/EBPα, CCAAT/enhancer binding protein alpha; PPARγ, peroxisome proliferator activated receptor γ; SREBP-1c, sterol regulatory element-binding protein-1C; Prdm16, PR domain containing 16; UCP1, uncoupling protein 1; PGC-1α, peroxisome proliferative activated receptor γ coactivator 1 α; GAPDH, glyceraldehyde-3-phosphate dehydrogenase.

**Table 2:** The body weight (b.w.), food intake, and fat weight of mice supplemented with phyllodulcin for 7 weeks. ^1^

	Ctrl	HF	SVS	P 20	P 40
Final b.w. (g)	35.56 ± 0.98 ^a^	43.73 ± 0.88 ^b^	43.58 ± 1.62 ^b^	43.59 ± 0.79 ^b^	43.95 ± 1.05 ^b^
BMI (kg/m^2^)	5.82 ± 0.18 ^a^	6.90 ± 0.14 ^b^	6.76 ± 0.14 ^b^	6.62 ± 0.26 ^b^	6.32 ± 0.10 ^ab^
Food intake (g/d)	3.23 ± 0.02 ^a^	2.92 ± 0.03 ^b^	2.68 ± 0.41 ^c^	2.81 ± 0.03 ^b^	2.83 ± 0.03 ^b^
Total fat mass of b.w. (%)	11.69 ± 0.77 ^a^	14.93 ± 0.39 ^b^	14.22 ± 0.40 ^b^	13.30 ± 0.99 ^ab^	13.24 ± 0.27 ^ab^
Subcutaneous fat (g)	1.16 ± 0.11 ^a^	2.11 ± 0.07 ^b^	1.88 ± 0.09 ^bc^	1.88 ± 0.20 ^bc^	1.63 ± 0.08 ^c^
Mesenteric fat (g)	0.67 ± 0.06 ^a^	1.21 ± 0.08 ^b^	1.32 ± 0.06 ^b^	1.15 ± 0.13 ^b^	1.17 ± 0.08 ^b^

^1^ The values shown are the mean ± standard error of the mean (SEM). Data were analyzed using one-way ANOVA with Tukey’s post hoc test (*p* < 0.05). Groups with a different letter statistically differ (*p* < 0.05). Ctrl, non-obese control; HF, high-fat diet-induced obese mice; SVS, high-fat diet-induced obese mice that received 40 mg/kg b.w. stevioside; P 20, high-fat diet-induced obese mice that received 20 mg/kg b.w. phyllodulcin; P 40, high-fat diet-induced obese mice that received 40 mg/kg b.w. phyllodulcin.

**Table 3:** The lipid profiles, fasting blood glucose, and leptin and adiponectin levels of mice supplemented with phyllodulcin for 7 weeks. ^1^

	Ctrl	HF	SVS	P 20	P 40
TG (mg/dL)	107.32 ± 5.51 ^a^	121.62 ± 4.91 ^b^	116.26 ± 3.10 ^ab^	104.68 ± 1.60 ^a^	103.76 ± 1.68 ^a^
TC (mg/dL)	106.87 ± 5.92 ^a^	132.89 ± 3.15 ^b^	113.46 ± 4.96 ^a^	113.00 ± 0.03 ^a^	113.66 ± 2.17 ^a^
LDL (mg/dL)	34.14 ± 3.64 ^a^	48.33 ± 2.70 ^b^	29.46 ± 2.71 ^a^	29.78 ± 5.19 ^a^	27.91 ± 3.15 ^a^
FBG (mg/dL)	111.30 ± 6.88 ^a^	208.18 ± 10.87 ^b^	137.50 ± 6.22 ^ac^	138.58 ± 5.63^c^	131.17 ± 3.54 ^ac^
Leptin (ng/mL)	18.18 ± 1.31 ^a^	27.47 ± 0.50 ^b^	25.95 ± 0.92 ^ab^	24.81 ± 1.54 ^ab^	22.63 ± 0.79 ^c^
Adiponectin (ng/mL)	1.10 ± 0.04 ^a^	0.81 ± 0.04 ^b^	0.82 ± 0.02 ^bc^	0.95 ± 0.04 ^cd^	1.00 ± 0.03 ^ad^

^1^ The values shown are the mean ± standard error of the mean (SEM). Data were analyzed using one-way ANOVA with Tukey’s post hoc test (*p* < 0.05). Groups with a different letter statistically differ (*p* < 0.05). Ctrl, non-obese control; HF, high-fat diet-induced obese mice; SVS, high-fat diet-induced obese mice that received 40 mg/kg b.w. stevioside; P 20, high-fat diet-induced obese mice that received 20 mg/kg b.w. phyllodulcin; P 40, high-fat diet-induced obese mice that received 40 mg/kg b.w. phyllodulcin; TG, triglyceride; TC, total cholesterol; LDL, low density lipoprotein; FBG, fasting blood glucose; b.w., body weight.
